# Prognostic significance of postoperative loss of skeletal muscle mass in patients underwent coronary artery bypass grafting

**DOI:** 10.3389/fnut.2022.970729

**Published:** 2022-09-02

**Authors:** Zi-Le Shen, Zhang Liu, Peng Zhang, Wei-Zhe Chen, Wen-Xi Dong, Wen-Hao Chen, Feng Lin, Wang-Fu Zang, Xia-Lin Yan, Zhen Yu

**Affiliations:** ^1^Department of Gastrointestinal Surgery, Shanghai Tenth People’s Hospital, Tongji University School of Medicine, Shanghai, China; ^2^Department of Cardio-Thoracic Surgery, Shanghai Tenth People’s Hospital, Tongji University School of Medicine, Shanghai, China; ^3^Department of Gastrointestinal Surgery, The First Affiliated Hospital of Wenzhou Medical University, Wenzhou, China; ^4^Department of Colorectal Anal Surgery, The First Affiliated Hospital of Wenzhou Medical University, Wenzhou, China

**Keywords:** skeletal muscle loss, coronary artery bypass grafting, nomogram, survival, oral nutritional supplement

## Abstract

**Background:**

Increasing life expectancy of coronary artery bypass grafting (CABG) remains to be the major concern of cardiac surgeons. However, few studies have investigated the effect of postoperative skeletal muscle index (SMI) loss on prognosis. This study aims to evaluate the prognostic role of postoperative SMI loss ≥ 5% after CABG, in order to develop a novel nomogram to predict overall survival (OS).

**Methods:**

Patients underwent CABG via midline sternotomy from December 2015 to March 2021 were recruited in this study. Preoperative and postoperative 3 months chest computed tomography (CT) images were compared to assess changes in SMI at T12 level. Based on this, patients were classified into the presence or absence of SMI loss ≥ 5%. The association between postoperative SMI loss ≥ 5% and OS was then analyzed by the Kaplan-Meier curves and Cox model. A novel nomogram incorporating independent clinical prognostic variables was also developed.

**Results:**

The study enrolled 506 patients receiving CABG, of whom 98 patients experienced T12 SMI loss ≥ 5% and had a significantly worse OS (*P* < 0.0001). Multivariate regression analysis showed that T12 SMI per cent change (%T12 SMI-change) was an independent prognostic factor for OS (HR = 0.809, 95% CI = 0.749–0.874). The nomogram incorporating %T12 SMI-change with other variables was accurate for predicting OS. Besides, we also found that postoperative oral nutritional supplement (ONS) can rescue T12 SMI loss.

**Conclusion:**

Postoperative SMI loss can predict survival outcome after CABG. The nomogram incorporating changes in SMI provides a superior performance than existing systems.

## Introduction

Coronary artery disease (CAD) is a global burden in terms of high mortality, morbidity and economic loss (1, 2). As a preferred treatment for patients with 3-vessel disease and left main CAD, coronary artery bypass grafting (CABG) provides surgical collateralization and prolong life (3, 4). However, preventing postoperative complications and increasing life expectancy still arouse cardiac surgeons’ concerns (5). Previous studies on improving the prognosis of patients underwent CABG have mainly focused on treatment-related factors, such as the selection of grafts, surgical approach methods, and the establishment of extracorporeal circulation (6–9). Conversely, some patient-related modifiable factors, such as postoperative weight loss, sarcopenia, etc., have not been studied extensively, and corresponding measures to improve these factors are lacking.

Measurement of body composition and its impact on outcomes after cardiac surgery are gaining increasing attention. Our study has previously confirmed that preoperative sarcopenia is associated with unfavorable short- and long-term prognosis of patients receiving CABG (10). Estimating preoperative skeletal muscle index (SMI) has a predictive effect and allows for timely therapeutic intervention for better postoperative outcomes. However, many patients underwent CABG experience muscle wasting during the recovery period, which results in reduced muscle mass, functional capacity and quality of life, and increased mortality, especially in patients older than 50 years (11, 12). Surgery-induced stress, inflammation, and protein depletion all contribute to disturbed metabolism and postoperative skeletal muscle loss (13, 14). Considering the dynamic change of SMI during perioperative period, it may inadequate to merely assess the state of skeletal muscle before surgery to predict the clinical outcomes of the patients and formulate postoperative rehabilitation programs. Nevertheless, the few existing studies mainly focused on the impact of preoperative skeletal muscle assessment on the prognosis of CABG patients (10, 15), and the relationship between postoperative skeletal muscle loss and clinical outcomes in these patients is unclear.

In this study, we explored the factors affecting SMI loss after CABG, and investigated the predictive role of postoperative SMI loss on long-term outcomes in CABG patients. The secondary objective was to develop a novel nomogram with body composition-related parameters for predicting overall survival (OS) and verify its predictive effect in patients underwent CABG.

## Materials and methods

### Patient eligibility and data collection

Between December 2015 and March 2021, patients aged ≥ 18 years who underwent CABG through midline sternotomy at the Department of Cardio-Thoracic Surgery, Shanghai Tenth People’s Hospital were recruited in this study. Patients with emergency surgery, a history of previous cardiothoracic surgery, insufficient chest computed tomography (CT) data, or in-hospital mortality were excluded. All the patients signed written informed consent. This study was conducted following the Helsinki Declaration of the World Medical Association, and the research approach was approved by the ethics committee of Shanghai Tenth People’s Hospital and registered in Chinese Clinical Trial Registry (ChiCTR2000037875).

For each patient, the following clinical data were collected by trained surgeons: (1) preoperative characteristics, including general information, cardiac function-related information, existing comorbidity, laboratory tests and nutritional risk assessment [evaluated by prognostic nutritional index (PNI) and geriatric nutritional risk index (GNRI)] during preoperative period; (2) operative features, including type of surgery, number of bypassed vessels, and operative time; (3) postoperative characteristics, including postoperative complications graded by Clavien-Dindo classification (16) (Grade ≥ II were analyzed), severe complications (Grade ≥ III) and the use of oral nutritional supplement (ONS). According to the doctor’s recommendation, patients with reduced eating were prescribed with Ruineng^®^ (Sino-Swed Pharmaceutical Corp. Ltd.) for 5 days after CABG. This ONS product is a nutritionally balanced enteral nutritional emulsion, containing approximately 650 kcal energy, 29.3 g protein, 36 g fat, 52 g of carbohydrate, and vitamins and minerals per 500 ml. Patients receiving the advice of physician took ONS after CABG, and the expected daily intake of ONS was 500 ml. And patients did not routinely receive exercise rehabilitation training after CABG.

### Quantification of skeletal muscle mass

The chest CT images performed within 2 weeks before and 3 months after CABG were collected, respectively. Then we analyzed these collected images at the 12th thoracic vertebra (T12) level by INFINITT PACS software (version 3.0.11.3, Seoul, Korea) to identify skeletal muscle in the range of –29 to + 150 Hounsfield unit (HU). Skeletal muscles evaluated at T12 level contained the rectus abdominis, external oblique, internal oblique, latissimus dorsi, intercostal, and erector spinae muscles. The skeletal muscle area was normalized by height (m^2^) to determine the SMI (cm^2^/m^2^) at T12. Consistent with our previous study, referring to a large-scale study, low SMI was defined as T12 SMI < 28.8 cm^2^/m^2^ for male and < 20.8 cm^2^/m^2^ for female (10, 17). T12 SMI per cent change (%T12 SMI-change) was calculated as (postoperative T12 SMI—preoperative T12 SMI)/preoperative T12 SMI × 100%. Since multiple studies have demonstrated that SMI loss ≥ 5% is associated with adverse clinical outcomes (18–21), we used this threshold to divide patients into T12 SMI loss ≥ 5% and T12 SMI loss < 5% groups to compare OS according to SMI change in this study.

### Follow-up

All patients were followed up every 3 months for the first 2 years, and every 6 months after that by telephone interviews or outpatient visits. OS was calculated from the date of operation until the date of death from any cause or the last follow-up date for live patients. The latest follow-up date was January 31, 2022.

### Statistical analysis

Depend on the normality of distribution, continuous variables were presented as the mean ± standard deviation (*SD*) and compared using the Student’s *t*-test, or median (p25–p75) and compared using the Mann-Whitney *U*-test or Kruskal-Wallis test. While categorical data were expressed as numbers (percentages), using Chi-squared or Fisher’s exact test for comparison. Univariate Cox regression analysis was performed to determine probable risk factors of OS. Factors with *P* < 0.10 were included in the multivariate Cox analysis by a backward stepwise selection methodology. Kaplan-Meier curves and Cox proportional hazards model were used to analyze long-term survival. The variables screened by multivariate Cox regression were incorporated to develop a nomogram for OS prediction. Random resampling of the study population with a 75% ratio was performed to simulate outsource validation cohort. C-index, the area under receiver operating characteristic curve (AUC) and calibration curve were performed to evaluated the discriminative ability and predictive accuracy of the novel nomogram. All tests were two-sided and *P* < 0.05 was regarded as statistically significant. All statistical analysis was conducted by SPSS software version 26.0 (Armonk, NY, United States) and R software version 4.1.3 (Vienna, Austria).

## Results

### Characteristics of the patients

**FIGURE 1:**
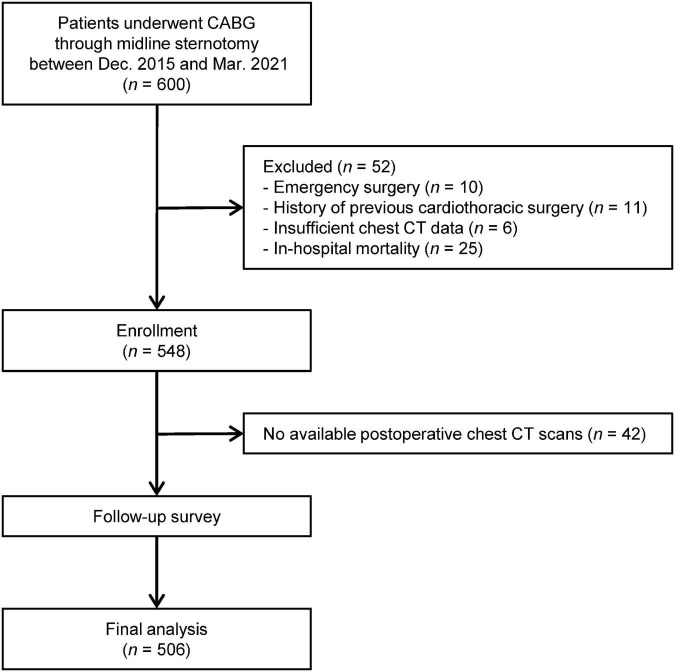
Flowchart of patient selection.

Initially, 548 patients underwent CABG *via* midline sternotomy met the inclusion criteria. And excluding unavailable postoperative chest CT scans in 42 patients, a total of 506 patients were finally analyzed in our study. The patient flow chart was shown in Figure 1.

Patient characteristics were presented in Table 1. Based on the preoperative and postoperative chest CT scans of each patient, 98 (19.37%) patients demonstrated T12 SMI loss ≥ 5%, while 408 (80.63%) patients had T12 SMI loss < 5%. The two groups were comparable in terms of age, gender, comorbidities, C-reactive protein, red blood cells, hemoglobin, neutrophil-to-lymphocyte ratio (NLR), albumin, blood urea nitrogen (BUN), creatinine, preoperative T12 SMI, surgical details, and postoperative complications. However, ≥ 5% T12 SMI loss was associated with lower body mass index (BMI) (*P* = 0.035) and postoperative T12 SMI (*P* < 0.001). In contrast, patients with T12 SMI ≥ 5% loss had higher white blood cells (*P* = 0.020). Although the left ventricular ejection (LVEF) and postoperative hospital stays of two groups were quite close, patients who exhibited ≥ 5% T12 SMI loss had higher LVEF (*P* = 0.045) and longer postoperative hospital stays (*P* = 0.045). For the risk score of cardiac surgery, EuroSCORE II score was higher in T12 SMI loss ≥ 5% group than in T12 SMI loss < 5% group (*P* = 0.023).

**TABLE 1:** Patient characteristics according to postoperative skeletal muscle mass loss.

	Total (*n* = 506)	T12 SMI loss		*P-value*
		≥5% (*n* = 98)	<5% (*n* = 408)	
Age, years	64.5 [59–70]^a^	65 [60.75–70]	64 [58–69]	0.228
Gender, male	384 (75.9)^b^	77 (78.6)	307 (75.2)	0.489
**Comorbidities**				
Hypertension	403 (79.6)	80 (81.6)	323 (79.2)	0.586
Diabetes	222 (43.9)	49 (50.0)	173 (42.4)	0.173
Previous MI	35 (6.9)	7 (7.1)	28 (6.9)	0.922
Cerebrovascular disease	75 (14.8)	15 (15.3)	60 (14.7)	0.881
Tobacco use, yes	201 (39.7)	43 (43.9)	158 (38.7)	0.349
Alcohol use, yes	91 (18.0)	21 (21.4)	70 (17.2)	0.323
**Laboratory data**				
C-reactive protein, mg/L	3.17 [3.02–5.62]	3.17 [3.02–6.53]	3.17 [3.02–5.11]	0.751
White blood cells, × 10^9^/L	6.66 [5.39–8.06]	7.06 [5.82–8.37]	6.50 [5.32–7.99]	0.020*
Red blood cells, × 10^12^/L	4.33 [3.97–4.73]	4.26 [3.96–4.67]	4.36 [3.97–4.75]	0.180
Hemoglobin, g/L	131 [119.75–143]	129 [118–141]	131.5 [120–144]	0.175
NLR	2.36 [1.72–3.41]	2.20 [1.59–3.44]	2.38 [1.75–3.40]	0.199
Albumin, g/L	41 [38.98–44]	41 [38–43]	41 [39–44]	0.331
BUN, μmol/L	5.81 [4.78–7.08]	6.00 [4.35–7.53]	5.80 [4.83–6.98]	0.929
Creatinine, μmol/L	76.00 [64.28–90.53]	77.95 [63.95–98.03]	75.65 [64.35–89.50]	0.219
BMI, kg/m^2^	24.69 [22.86–26.95]	24.01 [22.48–26.05]	24.91 [22.91–27.05]	0.035*
Preoperative T12 SMI, cm^2^/m^2^	33.03 [28.92–37.08]	32.82 [28.34–37.50]	33.03 [29.18–36.95]	0.518
Postoperative T12 SMI, cm^2^/m^2^	32.56 [28.83–37.00]	30.55 [25.92–34.89]	33.31 [29.49–37.93]	<0.001*
PNI				0.644
>45	421 (83.2)	80 (81.6)	341 (83.6)	
≤45	85 (16.8)	18 (18.4)	67 (16.4)	
GNRI				0.073
>98	447 (88.3)	82 (83.7)	365 (89.5)	
92–98	38 (7.5)	12 (12.2)	26 (6.4)	
82 to<92	17 (3.4)	2 (2.0)	15 (3.7)	
<82	4 (0.8)	2 (2.0)	2 (0.5)	
LVEF, %	60 [55-64]	60 [48-63.25]	60 [56-64]	0.045*
NYHA class 4	95 (18.8)	21 (21.4)	74 (18.1)	0.454
EuroSCORE II, %	1.66 [1.30-2.28]	1.91 [1.30-2.72]	1.63 [1.30-2.21]	0.023*
**Surgical details**				
Surgical type				0.405
Off-pump CABG	163 (32.2)	26 (26.5)	137 (33.6)	
On-pump CABG	294 (58.1)	62 (63.3)	232 (56.9)	
CABG + valve	49 (9.7)	10 (10.2)	39 (9.6)	
Use of LIMA	249 (49.2)	43 (43.9)	206 (50.5)	0.240
Number of bypassed vessels				0.361
1	41 (8.1)	12 (12.2)	29 (7.1)	
2	55 (10.9)	11 (11.2)	44 (10.8)	
3	154 (30.4)	26 (26.5)	128 (31.4)	
4 or more	256 (50.6)	49 (50.0)	207 (50.7)	
Operative time, min	220 [191–248.25]	218.5 [191.5–247]	220 [191–249]	0.788
CPB time, min	65.5 [0–89]	68.5 [0–88.75]	64.5 [0–89]	0.306
Postoperative complications	236 (46.6)	51 (52.0)	185 (45.3)	0.233
Severe complications	189 (37.4)	40 (40.8)	149 (36.5)	0.430
Postoperative hospital stays, days	10 [9–13]	10.5 [9–15]	10 [8–13]	0.045*

SMI, skeletal muscle index; MI, myocardial infarction; NLR, neutrophil-to-lymphocyte ratio; BUN, blood urea nitrogen; BMI, body mass index; PNI, prognostic nutritional index; GNRI, geriatric nutritional risk index; LVEF, left ventricular ejection; NYHA, New York Heart Association; EuroSCORE II, European System for Cardiac Operative Risk Evaluation II; CABG, coronary artery bypass grafting; LIMA, left internal mammary artery; CPB, cardiopulmonary bypass.

^a^Median [p25- p75], all such values.

^b^Number (percentage), all such values.

*Statistically significant (P < 0.05).

### The influence of various clinical factors on postoperative skeletal muscle index change

**FIGURE 2:**
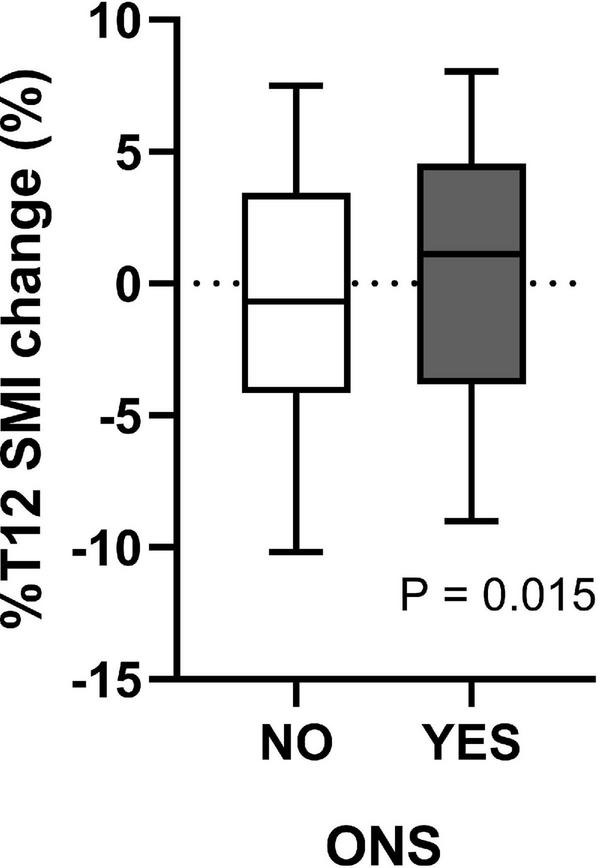
The effects of ONS on T12 SMI loss.

As shown in Supplementary Table 1, preoperative low SMI (*P* = 0.003), BMI (*P* = 0.023), LVEF (*P* = 0.013), hypoproteinemia (*P* < 0.001), PNI (*P* < 0.001), and GNRI (*P* = 0.013) may be essential preoperative factors affecting postoperative SMI change. However, postoperative complications did not affect postoperative %T12 SMI-change. In addition, of the 506 patients, 190 (37.5%) patients received doctor’s recommendation to take ONS after surgery. The median of %T12 SMI-change was –0.67 [–4.15–3.44]% and 1.12 [–3.82–4.54]% for patients without and with ONS, respectively (Figure 2). And there was significant difference in %T12 SMI-change between the two groups (*P* = 0.015).

### Relationship between postoperative skeletal muscle index loss and overall survival

**FIGURE 3:**
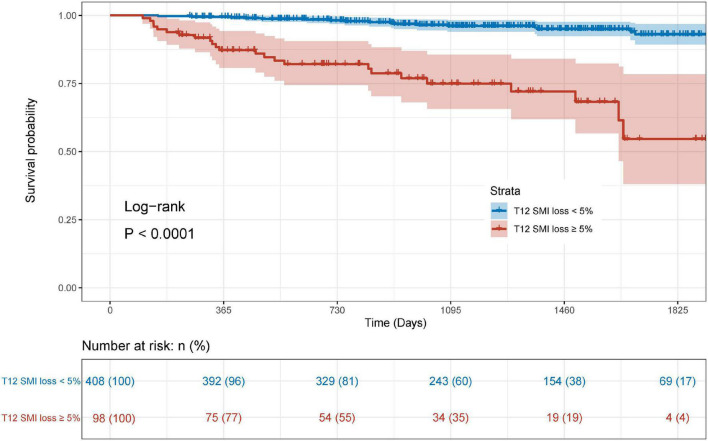
Kaplan-Meier curve for overall survival stratified by postoperative skeletal muscle mass loss.

During a median of 3.38 years follow-up, 43 (8.5%) patients died. The survival curves demonstrated that patients with T12 SMI ≥ 5% loss had a significantly worse OS relative to those with < 5% T12 SMI loss (Figure 3, Log-rank: *P* < 0.0001). Univariate and multivariate Cox regression analyses for OS were presented in Table 2. In the multivariate analysis, Preoperative low SMI (*P* = 0.003) and %T12 SMI-change (*P* < 0.001) were independent prognostic factor for OS in patients underwent CABG, alongside age (*P* = 0.010) and LVEF ≤ 50% (*P* = 0.033).

**TABLE 2:** Univariate and multivariate analyses for predictors of overall survival.

Factors	Univariate analysis		Multivariate analysis	
	HR (95% CI)	*P-value*	HR (95% CI)	*P-value*
Age, years	1.055 (1.018–1.095)	0.004*	1.051 (1.012–1.092)	0.010*
Gender, male	1.714 (0.763–3.853)	0.192		
Hypertension	0.991 (0.475–2.068)	0.981		
Diabetes	1.274 (0.700–2.319)	0.429		
Previous MI	1.031 (0.318–3.338)	0.959		
Cerebrovascular disease	1.796 (0.885–3.646)	0.105		
Tobacco use	1.318 (0.723–2.400)	0.367		
Alcohol use	1.282 (0.614–2.675)	0.508		
BMI, kg/m^2^	0.958 (0.868–1.057)	0.390		
Preoperative low SMI	4.379 (2.389–8.024)	<0.001*	2.638 (1.399–4.976)	0.003*
T12 SMI per cent change,%	0.790 (0.730–0.854)	<0.001*	0.809 (0.749–0.874)	<0.001*
LVEF ≤ 50%	2.374 (1.230–4.584)	0.010*	2.111 (1.063–4.190)	0.033*
NYHA class 4	1.718 (0.857–3.443)	0.127		
EuroSCORE II ≥ 4%	2.906 (1.219–6.929)	0.016*		
**Surgical type**				
Off-pump CABG	1 (Reference)	0.378		
On-pump CABG	1.453 (0.712–2.964)	0.304		
CABG + valve	1.962 (0.724–5.313)	0.185		
Use of LIMA	0.507 (0.268–0.960)	0.037*		
**Number of bypassed vessels**				
1	1 (Reference)	0.174		
2	0.872 (0.195–3.897)	0.857		
3	1.648 (0.487–5.572)	0.422		
4 or more	0.802 (0.235–2.741)	0.725		
Operative time, min	1.002 (0.996–1.007)	0.541		
CPB time, min	1.004 (0.999–1.010)	0.147		
C-reactive protein > 10 mg/L	1.175 (0.495–2.790)	0.715		
WBC > 10 × 10^9^/L	1.851 (0.726–4.719)	0.197		
NLR ≥ 3	1.270 (0.684–2.359)	0.449		
Hemoglobin, g/L	0.978 (0.962–0.994)	0.008*		
Albumin	0.970 (0.903–1.041)	0.396		
Postoperative ONS	0.818 (0.418–1.601)	0.557		

MI, myocardial infarction; BMI, body mass index; SMI, skeletal muscle index; LVEF, left ventricular ejection; NYHA, New York Heart Association; EuroSCORE II, European System for Cardiac Operative Risk Evaluation II; CABG, coronary artery bypass grafting; LIMA, left internal mammary artery; CPB, cardiopulmonary bypass; WBC, white blood cells; NLR, neutrophil-to-lymphocyte ratio; ONS, oral nutritional supplements.

*Statistically significant (P < 0.05).

### Construction and validation of the prognostic nomogram

**FIGURE 4:**
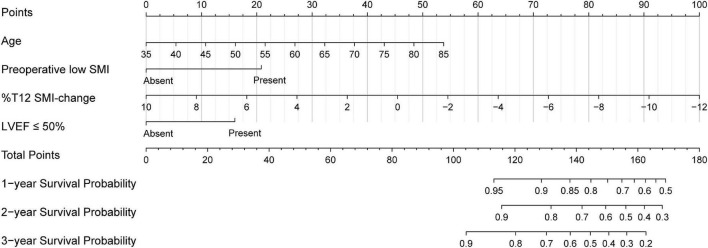
The nomogram developed to predict the overall survival. SMI, skeletal muscle index; %T12 SMI-change, T12 SMI percent change; LVEF, left ventricular ejection.

**FIGURE 5:**
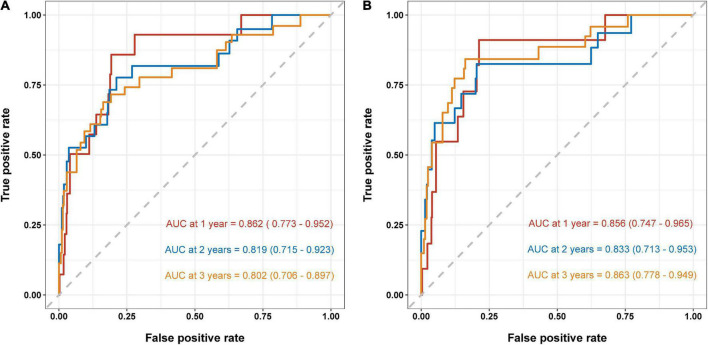
Area under the ROC curves (AUC) for survival prediction in the overall population **(A)** and validation cohort **(B)**. ROC, receiver operator characteristic.

**FIGURE 6:**
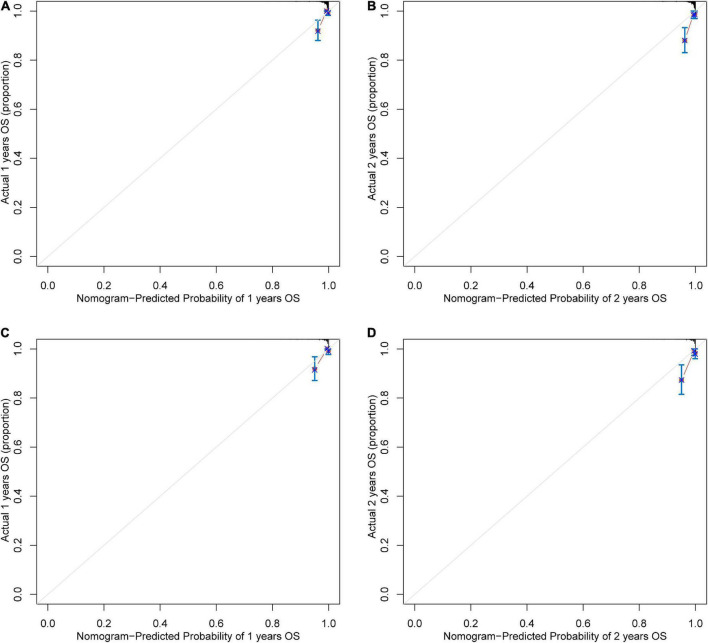
The calibration curve for survival prediction at **(A)** 1 year and **(B)** 2 years in the overall population and at **(C)** 1 year and **(D)** 2 years in the validation cohort. The nomogram-predicted probability of overall survival is plotted on the x-axis; the actual overall survival is plotted on the y-axis. OS, overall survival.

**FIGURE 7:**
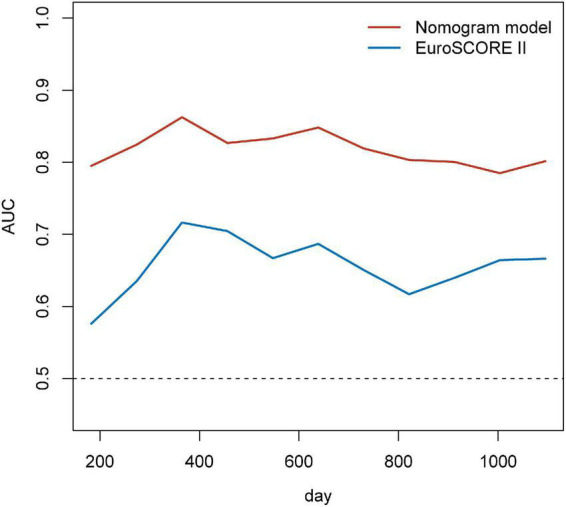
Time-dependent AUCs over time for EuroSCORE II and the nomogram for predicting the overall survival. EuroSCORE II, European System for Cardiac Operative Risk Evaluation II; AUC, area under the receiver operator characteristic curves.

Based on the results of multivariate Cox regression analysis, four independent predictors were integrated to developed a novel nomogram for predicting OS (Figure 4). Each variable has a score on the point scale, and the estimated probability of 1-, 2-, and 3-year OS could easily be obtained by adding the total score and placing it on the total score scale. Patient characteristics in validation cohort were shown in Supplementary Table 2. The C-index for overall and validation cohort were 0.77 (95% CI: 0.69-0.86) and 0.82 (95% CI: 0.72-0.92), respectively. Furthermore, the nomogram yielded AUC values of 0.862, 0.819, 0.802 in the overall population and 0.856, 0.833, 0.863 in the validation cohort for predicting OS rates at 1, 2 and 3 years (Figure 5). The calibration curve of the nomogram for the survival probability at 1 and 2 years demonstrated good agreement between prediction and observation in the overall population and the validation cohort (Figure 6). In addition, when compared with EuroSCORE II, the novel nomogram with body composition-related parameters demonstrated higher predictive ability of long-term survival (Figure 7).

## Discussion

To our knowledge, this is the first study to investigate the relationship between postoperative skeletal muscle loss and prognosis in patients underwent CABG. In the present study, we have demonstrated that about 20% of these patients have T12 SMI loss ≥ 5% 3 months after CABG, and postoperative ONS could rescue T12 SMI loss. Furthermore, we have presented evidence suggesting that %T12 SMI-change is an independent risk factor for OS of CABG. A nomogram incorporating %T12 SMI-change as well as the parameters such as age, preoperative low SMI, LVEF ≤ 50% was developed. Importantly, compared with EuroSCORE II, our novel nomogram showed stronger prediction efficiency for long-term outcomes.

Surgical trauma is frequently accompanied with hyperglycemia, accelerated systemic protein catabolism and amino acid oxidation, which results in nitrogen loss and negative protein balance (22, 23). A previous study has reported that the protein synthesis of skeletal muscle is severely inhibited after CABG surgery, with muscle protein synthesis rates decreasing by approximately 36% within the first 4 h after surgery (24). As an essential component of body composition, skeletal muscle mass (SMM) is most sensitive to changes in protein balance (25, 26). In the present study, 19.37% of patients experienced T12 SMI loss ≥ 5% at 3 months after CABG. In agreement with this finding, van Venrooij et al. also found that patients underwent cardiac surgery are more likely to suffer from skeletal muscle loss, especially in patients receiving CABG. Approximately 25% of those patients lose more than 5% of SMM within 2 months after surgery (18).

Among the several techniques to assess body composition, bioelectrical impedance analysis (BIA) is commonly used in primary care because of its portability and affordability and dual-energy X-ray absorptiometry (DXA) gain the popularity among some clinicians and researchers (27, 28). However, the muscle mass determined by BIA and DXA would sometimes fail to be consistent with each other due to the different bands of equipment and can be easily influenced by the body hydration status, such as the patients underwent CABG, among which fluid retention caused by unstable cardiac function often occurs (29). CT is the gold standard to assess muscle quantity/mass non-invasively (30). SMM at T12 level has good consistency with whole body muscle mass (17). Besides, the analysis based on the existing chest CT avoids extra radiation exposure and medical expenses of CABG patients. Based on the above considerations, chest CT was used to assess SMM in this study.

Recently, preoperative sarcopenia has become a prognostic indicator for various types of surgery (31–34), whereas few studies investigated the impact of preoperative skeletal muscle loss on survival in patients receiving CABG. In our previous study, preoperative sarcopenia was proved to be an independent risk factor for postoperative complications and OS of CABG (10). Actually, postoperative skeletal muscle loss not only leads to a decrease in postoperative quality of life (18), but also causes decreased exercise capacity and impaired respiratory function (35). Therefore, comparing with preoperative sarcopenia, postoperative skeletal muscle loss is more sensitive for worse long-term prognosis, as it reflects the response to the surgical trauma and stress of patients. In this study, patients with postoperative T12 SMI loss ≥ 5% were more likely to have adverse OS (Figure 3). Multivariate Cox analysis demonstrated that preoperative low SMI, %T12 SMI-change, age and LVEF ≤ 50% remain independent risk factors of OS. For patients underwent CABG, reduced LVEF is frequently associated with ischemic cardiomyopathy and increases the risk of postoperative adverse events (36). Thus, we incorporated all four independent risk factors into an innovative nomogram for predicting long-term survival in CABG patients.

EuroSCORE II, the most commonly used risk assessment tool, has been shown to be effective in predicting postoperative risk in patients underwent cardiac surgery (37, 38). And EuroSCORE II is mainly based on clinical characteristics, surgical factors, and echocardiographic findings (39). In addition to the above factors, inflammation, left ventricular systolic dysfunction, nutritional status, and body composition can also affect patient outcomes. Even though EuroSCORE II is accurate in predicting short-term outcomes, its performance fades for mortality at follow-up longer than 30 days (40, 41). Therefore, it is necessary to develop a new risk stratification tool to predict long-term clinical outcomes. In this study, we exploited a nomogram for predicting OS in CABG patients, which showed favorable discrimination with AUC values consistently more than 0.8 and significantly higher than EuroSCORE II. The calibration curve of our nomogram for the survival probability at 1- and 2-year revealed good agreement between predicted and actual OS.

Exercise-based cardiac rehabilitation has been strongly recommended as an important adjunctive therapy after CABG in recent years (42, 43). Previous studies have demonstrated that exercise rehabilitation training can reduce muscle strength loss and improve postoperative quality of life after CABG (44–46). Whereas, some factors, such as postoperative pain, the risk of exercise, and the lack of local physiotherapy services make exercise-based cardiac rehabilitation programs less effective. The use of ONS can provide supplementary energy and nutrition in addition to normal food to improve energy expenditure from surgical trauma. Multiple previous studies have demonstrated that ONS can benefit patients by reducing postoperative skeletal muscle loss in many surgical procedures (47–49). As expected, ONS alleviated postoperative SMI loss in patients underwent CABG in our study, which adds a new strategy for cardiac rehabilitation programs. And as a reflection of systemic nutritional status, changes in SMI can be considered for evaluating the effects of ONS and guiding dietary recommendations.

There are some limitations to the present study. This is a single-center study and its findings need to be validated in international multi-center studies. Nonetheless, the present study still presents a large impact of postoperative SMI loss on OS. Else, due to the retrospective design of the study cohort, we only investigated changes in SMI in this study, and our future prospective studies intend to focus on the changes in skeletal muscle density and function after cardiac surgery. Finally, we only found that the application of ONS was associated with improved postoperative skeletal muscle loss in this study, and we are conducting a prospective study to further investigate the effect of ONS on clinical outcomes.

## Conclusion

Postoperative SMI loss has an effective prognostic influence on long-term survival after CABG. ONS may be considered for patients underwent cardiac surgery to reduce skeletal muscle degradation and improve outcomes. The nomogram incorporating changes in SMI performs well in survival prediction.

## Data availability statement

The raw data supporting the conclusions of this article will be made available by the authors, without undue reservation.

## Ethics statement

The studies involving human participants were reviewed and approved by the Ethics Committee of Shanghai Tenth People’s Hospital. The patients/participants provided their written informed consent to participate in this study.

## Author contributions

Z-LS, W-FZ, X-LY, and ZY designed the study. PZ, W-ZC, W-XD, W-HC, and FL collected the data. ZL did the analysis and interpretation of data. Z-LS wrote the article. ZY revised the article and took the decision to submit the article for publication. All authors gave final approval and agreed to take responsibility for all aspects of this work to ensuring integrity and accuracy.
