# Supplementary material for: Prognostic significance of postoperative loss of skeletal muscle mass in patients underwent coronary artery bypass grafting
Source: Front Nutr. 2022 Sep 2;9:970729. doi: 10.3389/fnut.2022.970729 (PMC9478409; doi:10.3389/fnut.2022.970729)
Supplement: Supplementary file 1 [file Data_Sheet_1.docx]

Supplementary Material

**Supplementary Table 1**

Postoperative %SMI change for each clinical factor

|  | %T12 SMI-change | P value |
| --- | --- | --- |
| Age, years |  | 0.483 |
| ≥ 65 / < 65 | -0.29 [-4.41─4.06] / 0.19 [-3.97─4.02] ^a^ |  |
| Gender |  | 0.138 |
| Male / Female | -0.41 [-4.41─3.93] / 0.76 [-3.23─4.31] |  |
| Preoperative low SMI |  | 0.003^*^ |
| Yes / No | -1.42 [-6.35─2.66] / 0.24 [-3.90─4.11] |  |
| BMI, kg/m^2^ |  | 0.023^*^ |
| < 18.5kg/m^2^ | -4.06 [-6.19─-1.69] |  |
| 18.5 to < 24kg/m^2^ | -0.29 [-4.46─3.68] |  |
| 24 to < 28kg/m^2^ | -0.14 [-4.15─4.05] |  |
| ≥ 28kg/m^2^ | 1.10 [-2.91─4.89] |  |
| LVEF, % |  | 0.013^*^ |
| > 50 / ≤ 50 | 0.05 [-3.79─4.11] / -1.29 [-6.12─2.59] |  |
| NYHA class 4 |  | 0.063 |
| Yes / No | -1.17 [-4.82─3.01] / 0.23 [-3.91─4.09] |  |
| Postoperative complications |  | 0.732 |
| Yes / No | -0.26 [-4.63─4.20] / -0.09 [-3.85─3.83] |  |
| Severe complications |  | 0.560 |
| Yes / No | 0.25 [-4.63─4.32] / -0.29 [-4.03─3.81] |  |
| C-reactive protein > 10 mg/L |  | 0.304 |
| Yes / No | 0.45 [-3.16─4.83] / -0.26 [-4.37─3.90] |  |
| WBC > 10 ×10^9^/L |  | 0.054 |
| Yes / No | -1.33 [-5.97─1.66] / 0.09 [-4.03─4.04] |  |
| NLR ≥ 3 |  | 0.473 |
| Yes / No | -0.39 [-3.95─3.70] / 0.18 [-4.13─4.19] |  |
| Postoperative ONS |  | 0.015^*^ |
| Yes / No | 1.12 [-3.82─4.54] / -0.67 [-4.15─3.44] |  |
| Hypoproteinemia ^b^ |  | < 0.001^*^ |
| Yes / No | -3.20 [-6.65─-0.31] / 0.19 [-3.94─4.16] |  |
| PNI |  | < 0.001^*^ |
| ≤ 45 / > 45 | -2.20 [-4.73─0.76] / 0.44 [-3.95─4.36] |  |
| GNRI |  | 0.013^*^ |
| > 98 | 0.25 [-3.93─4.12] |  |
| 92 - 98 | -1.37 [-6.65─3.38] |  |
| 82 to < 92 | -3.20 [-4.59─-0.72] |  |
| < 82 | -3.28 [-6.83─1.59] |  |

SMI, skeletal muscle index; BMI, body mass index; LVEF, left ventricular ejection; NYHA, New York Heart Association; WBC, white blood cells; NLR, neutrophil-to-lymphocyte ratio; ONS, oral nutritional supplements; PNI, prognostic nutritional index; GNRI, geriatric nutritional risk index.

^a^ Median [p25─p75], all such values.

^b^ Albumin < 35 g/L was defined as hypoproteinemia.

^*^ Statistically significant (P < 0.05).

**Supplementary Table 2**

Patient characteristics in validation cohort

|  | Total  (n = 380) |
| --- | --- |
|  |  |
| Age, years | 65 [60─70] ^a^ |
| Gender, male | 286 (75.3) ^b^ |
| Comorbidities |  |
| Hypertension | 302 (79.5) |
| Diabetes | 166 (43.7) |
| Previous MI | 26 (6.8) |
| Cerebrovascular disease | 55 (14.5) |
| Tobacco use, yes | 147 (38.7) |
| Alcohol use, yes | 63 (16.6) |
| Laboratory data |  |
| C-reactive protein, mg/L | 3.17 [3.02─6.29] |
| White blood cells, ×10^9^/L | 6.62 [5.41─8.00] |
| Red blood cells, ×10^12^/L | 4.32 [3.97─4.73] |
| Hemoglobin, g/L | 131 [119─143] |
| NLR | 2.41 [1.74─3.38] |
| Albumin, g/L | 41 [39─44] |
| BUN, μmol/L | 5.85 [4.80─7.10] |
| Creatinine, μmol/L | 76.00 [64.23─91.1] |
| BMI, kg/m^2^ | 24.69 [22.89─26.89] |
| Preoperative T12 SMI, cm^2^/m^2^ | 33.05 [29.00─37.21] |
| Postoperative T12 SMI, cm^2^/m^2^ | 32.56 [28.98─36.90] |
| PNI |  |
| > 45 | 316 (83.2) |
| ≤ 45 | 64 (16.8) |
| GNRI |  |
| > 98 | 338 (88.9) |
| 92 ─ 98 | 30 (7.9) |
| 82 to < 92 | 10 (2.6) |
| < 82 | 2 (0.5) |
| LVEF, % | 60 [55─64] |
| NYHA class 4 | 64 (16.8) |
| EuroSCORE II, % | 1.69 [1.29─2.31] |
| Surgical details |  |
| Surgical Type |  |
| Off-pump CABG | 118 (31.1) |
| On-pump CABG | 227 (59.7) |
| CABG + valve | 35 (9.2) |
| Use of LIMA | 186 (48.9) |
| Number of bypassed vessels |  |
| 1 | 27 (7.1) |
| 2 | 38 (10.0) |
| 3 | 116 (30.5) |
| 4 or more | 199 (52.4) |
| Operative time, min | 222.5 [193.5─250] |
| CPB time, min | 66 [0─89.75] |
| Postoperative complications | 172 (45.3) |
| Severe complications | 137 (36.1) |
| Postoperative hospital stays, days | 10 [9─13] |

SMI, skeletal muscle index; MI, myocardial infarction; NLR, neutrophil-to-lymphocyte ratio; BUN, blood urea nitrogen; BMI, body mass index; PNI, prognostic nutritional index; GNRI, geriatric nutritional risk index; LVEF, left ventricular ejection; NYHA, New York Heart Association; EuroSCORE II, European System for Cardiac Operative Risk Evaluation II; CABG, coronary artery bypass grafting; LIMA, left internal mammary artery; CPB, cardiopulmonary bypass.

^a^ Median [p25─p75], all such values.

^b^ Number (percentage), all such values.
